# Sustained hyperkalemia in an asymptomatic primary care patient. When to suspect familial pseudohyperkalemia

**DOI:** 10.1515/almed-2022-0057

**Published:** 2022-08-26

**Authors:** Carlos Castillo Pérez, Laura Rodríguez Alonso, Adrián Prados Boluda, Marta Cebrián Ballesteros, Blanca Torrubia Dodero

**Affiliations:** Médico adjunto de Análisis Clínicos. Departamento de bioquímica general, hormonas y marcadores tumorales, Servicio de Bioquímica Clínica. Hospital Universitario Fundación Jiménez-Díaz, Madrid, Spain; Química adjunta de Análisis Clínicos. Departamento de bioquímica general, hormonas y marcadores tumorales, Servicio de Bioquímica Clínica. Hospital Universitario Fundación Jiménez-Díaz, Madrid, Spain; Médico residente de segundo año. Departamento de bioquímica general, hormonas y marcadores tumorales, Servicio de Bioquímica Clínica. Hospital Universitario Fundación Jiménez-Díaz, Madrid, Spain; Farmacéutica adjunta de Análisis Clínicos. Departamento de bioquímica general, hormonas y marcadores tumorales, Servicio de Bioquímica Clínica. Hospital Universitario Fundación Jiménez-Díaz, Madrid, Spain

**Keywords:** incubation, potassium, pseudohyperkalemia

## Abstract

**Objectives:**

Study and management of a case with elevated potassium levels without apparent clinical causes in successive follow-up visits.

**Case presentation:**

We present the case of a primary care female patient who persistently exhibited elevated levels of potassium (5.3–5.9 mmol/L) in successive control laboratory tests, without an apparent clinical cause. The patient was ultimately referred to the Unit of Nephrology, where a potassium-low diet was indicated. Diet did not have any effect on potassium levels. After a thorough study, the cause of hyperkalemia could not be determined.

**Conclusions:**

The inconsistency between elevated potassium levels and the reason of consultation, and exclusion of other pre-analytical or pathological causes raised suspicion of familial pseudohyperkalemia. The sample was incubated at different times and temperatures to demonstrate their influence on levels of potassium in blood. Familial pseudohyperkalemia was established as the most probable diagnosis. Finally, the patient was discharged from the Unit of Nephrology and instructed to follow a normal diet.

## Introduction

**Figure 1:**
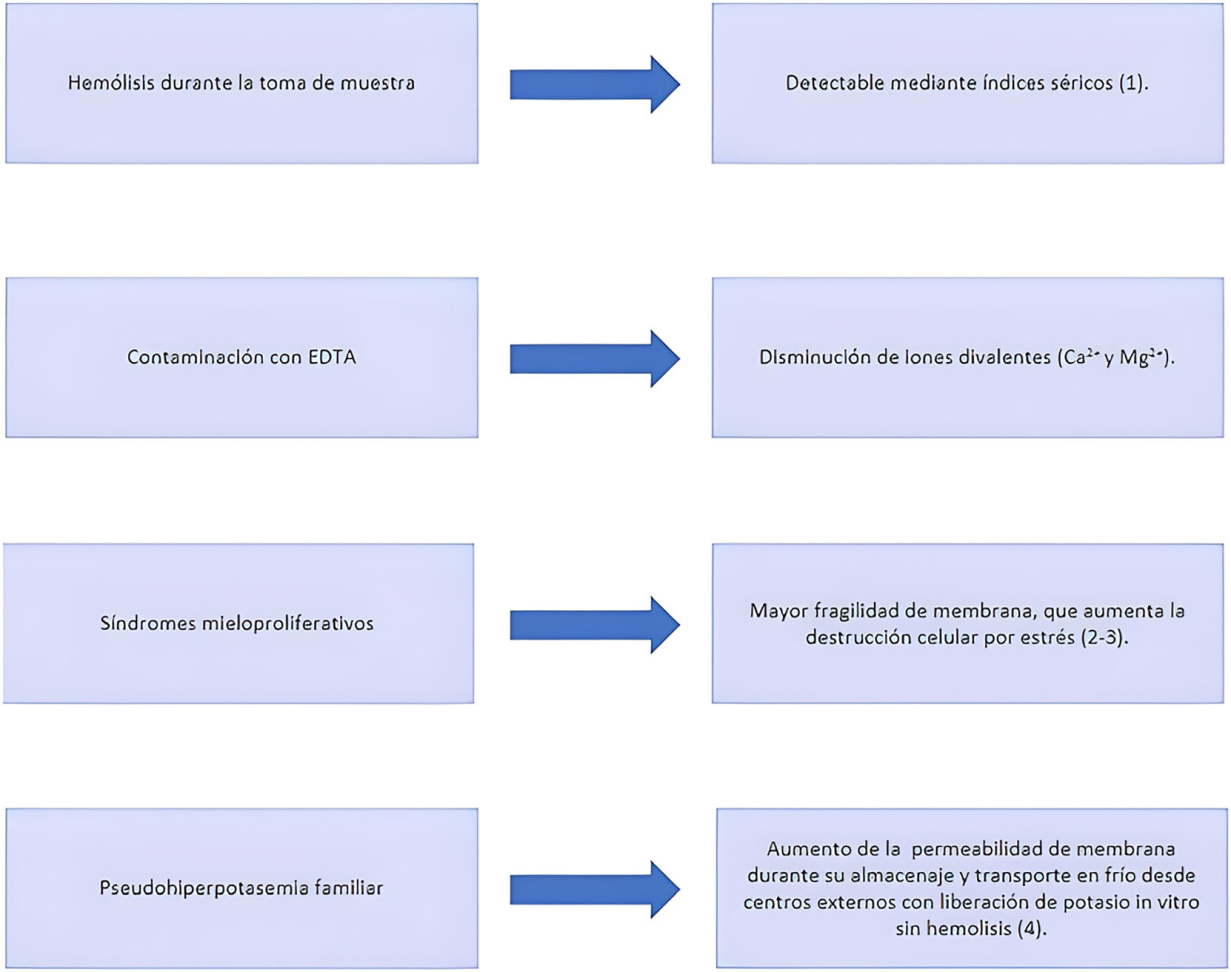
Most frequent causes of pseudohyperkalemia.

Elevated levels of potassium in blood may have deleterious effects on human health, including arrhythmias, muscle weakness, or paralysis [1]. Meeting pre-analytical and analytical standards is crucial for the diagnosis and monitoring of potassium-related diseases. Pseudohyperkalemia is defined as falsely raised potassium concentrations *in vitro* that are not consistent with real concentrations in the body. The causes of pseudohyperkalemia are described in Figure 1.

There is a clinical entity known as familial pseudohyperkalemia (FP). In FP, the patency of red cell membrane increases with *in vitro* refrigeration at 4 °C, which results in an elevation of potassium levels.

It is crucial that laboratories establish a protocol to detect cases of potassium elevation without associated clinical symptoms, where the most common causes of pseudohyperkalemia are excluded.

## Case presentation

We present the case of a 57 year-old woman on follow-up in primary care for a four-year history of mild hyperkalemia [range 5.3–5.9 mmol/L (reference range: 3.5–5.1 mmol/L)] without associated hemolysis (spectrophotometric hemolysis index <6 mg/dL in all laboratory reports). The patient did not exhibit any associated signs or symptoms. Control electrocardiograms were normal, without significant findings. Her medical history included arterial hypertension, adequately controlled with ACE inhibitors. In this context, the patient was referred to the Unit of Nephrology for further examination.

Anamnesis and physical examination did not reveal any data of interest. The patient was receiving amlodipine for arterial hypertension, with good control. Control electrocardiogram was normal. A potassium-low diet and control laboratory analysis in three months were indicated. Three months later, a control blood analysis was performed. The laboratory received a sample of serum and a request for determination of potassium alone. The sample was not hemolyzed (hemolysis index <6 mg/dL) and showed mild hyperkalemia of 5.5 mmol/L. Once pre-analytical and drug-related causes were excluded, differential diagnosis was performed, including:

1. Hyperkalemia secondary to renal insufficiency.
2. Hyperkalemia secondary to intravascular hemolysis.
3. Hyperkalemia secondary to acidosis.
4. Hyperkalemia secondary to tissue breakdown (rhabdomyolysis).
5. Pseudohyperkalemia secondary to oncohematologic disease (essential thrombocytosis, acute or chronic leukemia).

For differential diagnosis in the laboratory, blood was drawn at the hospital and sent to the laboratory with the following tests request:

– Hemogram including reticulocytes and blood smear.
– General biochemistry including ions, hemolysis index, hepatic profile (GOT, GPT, GGT, LDH, and ALP), renal profile (creatinine, urea, and estimated glomerular filtration rate CKD-EPI), creatine kinase, haptoglobin, phosphocalcic, and iron metabolism (iron, transferrin saturation index, transferrin, and ferritin).
– Venous gasometry: Blood gas test is immediately performed with a pH of 7.39 (range of reference 7.35–7.45).
– 24-urine biochemistry.

An EDTA sample was sent to obtain a hemogram and a lithium heparin sample for biochemistry. Smear was normal, without morphological alterations. All parameters in the hemogram were within normal range, with 1.55% reticulocytes (range of reference: 0.9–2.6%). Oncohematologic diseases were excluded by differential diagnosis.

The only finding on general biochemistry was cholesterol 248 mg/dL (range of reference: <200 mg/dL), with potassium 3.98 mmol/L (range of reference in plasma: 3.4–4.5 mmol/L), creatinine 0.86 mg/dL (range of reference: 0.51–0.95 mg/dL), and haptoglobin 39 mg/dL (range of reference: 30–200 mg/dL), and a hemolysis index <6 mg/dL. PCR, CK, hepatic profile and other parameters were within normal range. Laboratory analysis excluded hyperkalemia, due to the absence of renal insufficiency or data of cellular lysis or signs of intravascular hemolysis. Urine biochemistry was normal, with all parameters within normal range.

Once all potential causes were excluded by differential diagnosis, and in the absence of elevated potassium levels, a possible case of familial pseudohyperkalemia was considered. Three lithium heparin tubes were collected at the hospital for incubation at different time points and temperatures:

1. Two aliquots for incubation at 4 °C for 2 and 4 h, respectively. This simulated transport conditions from the primary care center to the laboratory, since samples are transported at 4 °C for a mean of 3 h.
2. Two aliquots for incubation at 25 °C for 2 and 4 h, respectively. This simulates transport conditions from the room where blood is drawn to the laboratory, since samples are transported at room temperature for a mean of 3 h.
3. Two aliquots for incubation at 37 °C for 2 and 4 h, respectively. This simulates *in vivo* conditions.

**Figure 2:**
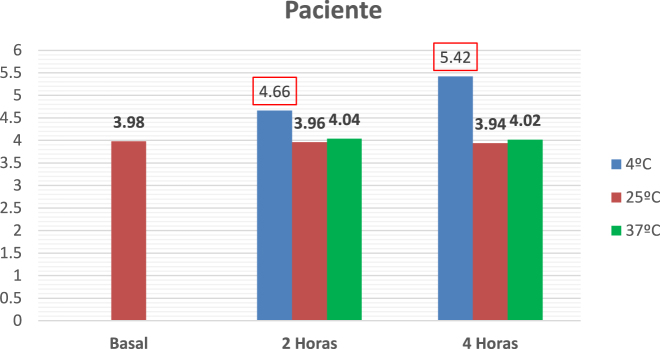
Sample of the patient. Potassium concentrations in the sample after incubation at different time points and temperatures. Red: Values exceeding the range of reference.

**Figure 3:**
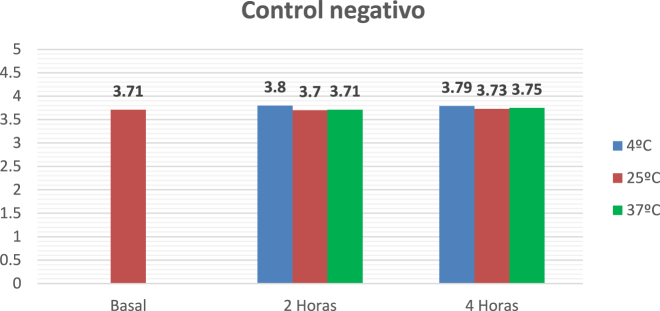
Negative control sample. Potassium concentrations after incubation at different time points and temperatures.

Uncentrifuged samples were received for analysis. Centrifugation was performed after the incubation period was completed (results shown in Figure 2). In parallel, a negative control was performed (Results shown in Figure 3).

## Discussion

Electrocardiographic and neurological alterations are early manifestations of hyperkalemia [2]. These manifestations originate from elevated tissue excitability, which may lead to the development of malignant arrhythmias (complex tachycardias or ventricular fibrillation) and neurological symptoms (muscle cramps, paralysis or paresthesias) [2]. These alterations suggest a strong correlation between potassium levels and electrocardiographic changes [3].

In this case, the main organic causes of hyperkalemia were ruled out, since no analytical alterations were observed. The patient did not develop hemolysis or other less frequent causes of pseudohyperkalemia, such as oncohematological syndromes. Plasma potassium concentrations were normal when blood was drawn at the hospital and elevated when the blood sample was transferred at 4 °C from the primary care center to the laboratory, which raised suspicion of a possible FP.

**Figure 4:**
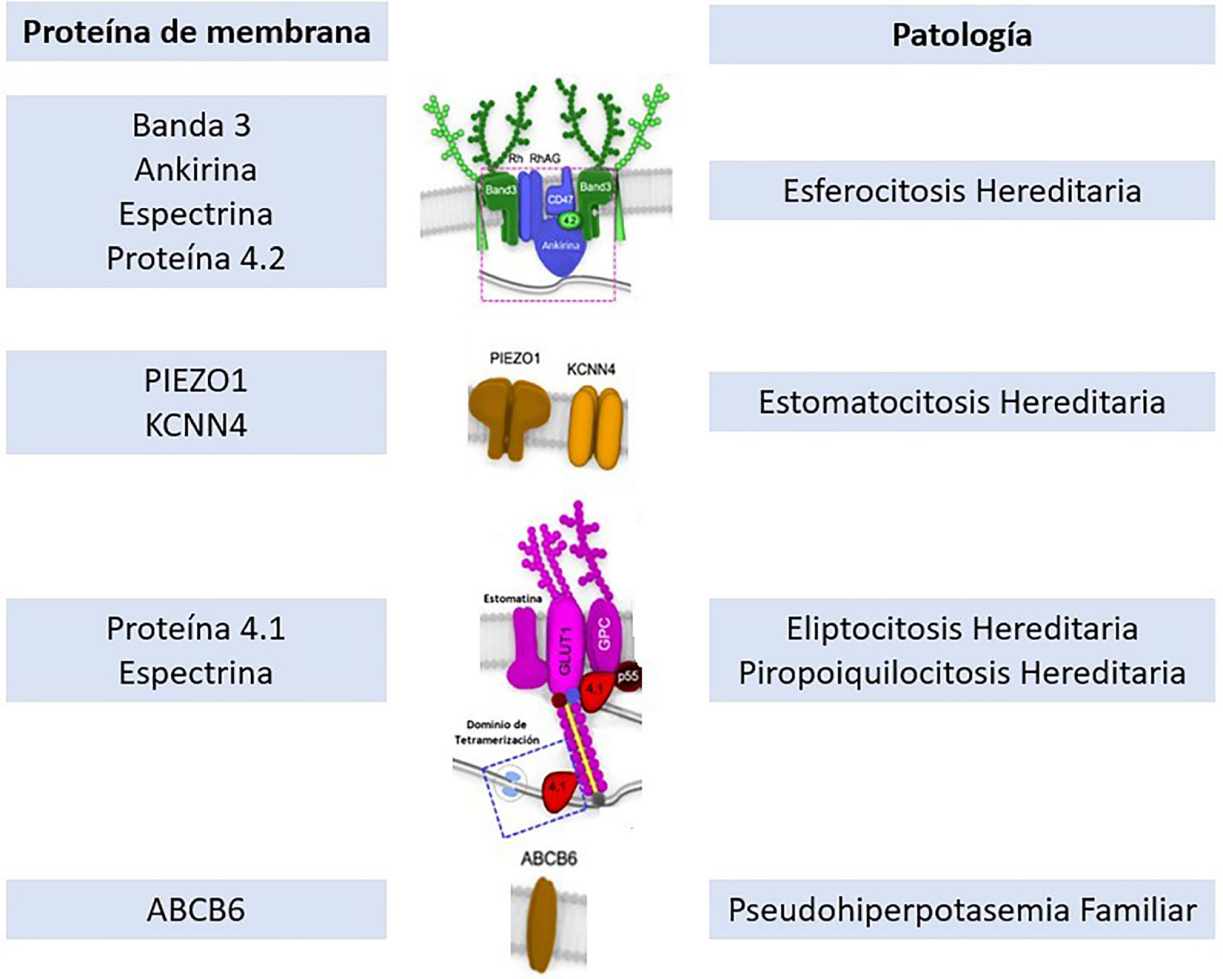
Diagram of erythrocyte membrane proteins with their associated disorders. Based on: Yonggoo K, Joonhong P, Myungshin K. Diagnostic approaches for inherited hemolytic anemia in the genetic era. Blood Res. 2017 Jun; 52 (2): 84–94.

FP belongs to the family of erythrocyte membrane disorders, which are classified into different types (Figure 4):

1. Hemolytic anemia secondary to membrane defects, with absence of patency alterations. Inherited spherocytosis, pyropoikilocytosis, and elliptocytosis belong to this group.
2. Hemolytic anemia due to membrane defects, with patency alterations. Hereditary stomatocytosis is an example of this group of disorders.
3. Membrane defects with patency alterations, with absence of anemia. FP belongs to this group.

FP originates from a mutation in the 2q35-q36 region of the *ABCB6* gene, with autosomal dominant inheritance [4]. Morphological alterations are rarely found on smear. In FP, potassium is elevated when the sample is stored at low temperatures (<8–10 °C), and normal when stored at temperatures >25 °C [5]. The ABCB6 protein is a membrane ATPase initially identified as a porphyrin transporter, although its functions are still unclear. Known mutations do not cause synthesis reduction, but a gain in transporter function [5]. This leads to a loss of potassium in samples refrigerated at low temperatures.

It is important that patients are recommended not to donate blood. Refrigeration of packed RBCs results in a release of potassium. There are reported cases of adverse events and even death after a RBC transfusion in patients with FP [6–8].

In view that potassium levels were persistently elevated without a known cause, the patient was referred to the Unit of Nephrology. A potassium-low diet was established, without it having any effects on potassium levels. When diet fails and an organic cause is not detected, FP must be suspected. An assay involving the incubation of aliquots helps simulate pre-analytical conditions in centers of reference. This method is useful to compare the behavior of RBC patency *in vitro* and determine whether the cause of hyperkalemia is FP. It is recommended that final diagnosis is established by *ABCB6* gene sequencing to detect mutations known to be associated with this disorder.

## Conclusions

When the clinical status of a patient is not consistent with elevated levels of potassium, and/or indications and treatments to reduce kalemia are unsuccessful, a request must be sent for the clinical laboratory to study hyperkalemia from different clinical and methodological approaches, to establish a possible diagnosis of FP. This is crucial for treatments to be effective and for the quality of life of our patients.
